# Imported submicroscopic malaria in Madrid

**DOI:** 10.1186/1475-2875-11-324

**Published:** 2012-09-12

**Authors:** Germán Ramírez-Olivencia, José Miguel Rubio, Pablo Rivas, Mercedes Subirats, María Dolores Herrero, Mar Lago, Sabino Puente

**Affiliations:** 1 Unit of Tropical Medicine, Infectious Disease Department, Hospital Carlos III, 1028029, Madrid, Spain; 2 Malaria & Emerging Parasitic Diseases Laboratory, Parasitology Department, National Centre of Microbiology, Instituto de Salud Carlos III (ISCIII), 28220, Madrid, Spain; 3 Microbiology and Parasitology Department, Hospital Carlos III, 1028029, Madrid, Spain

**Keywords:** Submicroscopic malaria, Paludism, Asymptomatic malaria, Traveller, Immigrants, PCR

## Abstract

**Background:**

Submicroscopic malaria (SMM) can be defined as low-density infections of *Plasmodium* that are unlikely to be detected by conventional microscopy. Such submicroscopic infections only occasionally cause acute disease, but they are capable of infecting mosquitoes and contributing to transmission. This entity is frequent in endemic countries; however, little is known about imported SMM.

The goals of this study were two-fold: a) to know the frequency of imported SMM, and b) to describe epidemiological, laboratorial and clinical features of imported SMM.

**Methods:**

A retrospective study based on review of medical records was performed. The study population consisted of patients older than 15 years attended at the Tropical Medicine Unit of Hospital Carlos III, between January 1, 2002 and December 31, 2007. Routinely detection techniques for *Plasmodium* included Field staining and microscopic examination through thick and thin blood smear. A semi-nested multiplex malaria PCR was used to diagnose or to confirm cases with low parasitaemia.

**Results:**

SMM was diagnosed in 104 cases, representing 35.5% of all malaria cases. Mean age (IC95%) was 40.38 years (37.41-43.34), and sex distribution was similar. Most cases were in immigrants, but some cases were found in travellers. Equatorial Guinea was the main country where infection was acquired (81.7%). Symptoms were present only in 28.8% of all SMM cases, mainly asthenia (73.3% of symptomatic patients), fever (60%) and arthromialgias (53.3%). The associated laboratory abnormalities were anaemia (27.9%), leukopaenia (15.4%) and thrombopaenia (15.4%). Co-morbidity was described in 75 cases (72.1%).

**Conclusions:**

Results from this study suggest that imported SMM should be considered in some patients attended at Tropical Medicine Units. Although it is usually asymptomatic, it may be responsible of fever, or laboratory abnormalities in patients coming from endemic areas. The possibility of transmission in SMM has been previously described in endemic zones, and presence of vector in Europe has also been reported. Implementation of molecular tests in all asymptomatic individuals coming from endemic area is not economically feasible. So re-emergence of malaria (*Plasmodium vivax*) in Europe may be speculated.

## Abstract (es)

**Abstract in Spanish:**

**Introducción**

La malaria submicroscópica (MSM) puede ser definida como una infección por *Plasmodium* de baja densidad, no detectable mediante microscopía convencional. Ocasionalmente produce enfermedad aguda, pero es capaz de producir infección el mosquito y ser transmitida. Es frecuente en regiones endémicas; sin embargo, existe poca información sobre la MSM importada.

El objetivo de este estudio es doble: a) Conocer la frecuencia de MSM importada; b) Describir las caracetrísitcas epidemiológicas, analíticas y clínicas de la MSM importada.

**Métodos**

Estudio retrospectivo mediante revisión de historias clínicas. La población de estudio estaba formada por mayores de 15 años atendidos en la Unidad de Medicina Tropical del Hospital Carlos III, entre el 1 de enero de 2002 y el 31 de diciembre de 2007. Las técnicas rutinarias de detección de *Plasmodium* incluyen tinción de Field, gota gruesa y extensión. Una técnica de PCR (seminested multiplex) fue utilizada para el diagnostico o para confirmar casos con baja parasitemias.

**Resultados**

La MSM fue diagnosticada en 104 casos, representando el 35.5% de los casos de malaria. La edad media (IC95%) fue 40.38 años (37.41-43.34), y la distribución por sexos fue similar. La mayoría de los casos eran en inmigrantes, describiéndose también casos en viajeros. Guinea Ecuatorial fue el principal país de adquisición de la infección (81.7%). Los síntomas estaban presentes en el 28.8% de todos los casos de MSM, principalmente astenia (73.3% de los pacientes sintomáticos), fiebre (60%) y artromialgias (53.3%). Las anomalías analíticas asociadas fueron anemia (27.9%), leucopenia (15.4%) y trombopenia (15.4%). Había comorbilidad en 75 casos (72.1%).

**Discusión**

Estos resultados sugieren que debería considerarse la MSM importada en algunos pacientes atendidos en las Unidades de Medicina Tropical. Aunque suele ser asintomática, puede ser responsable de fiebre o alteraciones analíticas en provenientes de regiones endémicas. La posibilidad de transmisión de la MSM ha sido previamente demostrada, así como la presencia en Europa del vector. La implementación de tests moleculares a todos los individuos asintomáticos que provienen de regiones endémicas no es económicamente factible. Por tanto, se puede especular con la idea de la re-emergencia de malaria en Europa (al menos de *P. vivax*).

## Background

Malaria is usually diagnosed using microscopic methods (thick or thin blood smears) or immunochromatography. These procedures are routinely used in clinical practice, but have limited sensitivity. Submicroscopic malaria (SMM) is defined as low-density infections of *Plasmodium* that are unlikely detected by conventional microscopy.

Several questions arise from this definition: Is SMM a real entity or “only” a laboratory finding? Is SMM a rare condition or a frequently neglected problem? And finally, can SMM play a role in malaria transmission or is it a problem only for individual patients? Since the development of polymerase chain reaction (PCR) techniques, attempts have been made to answer these questions. SMM has been associated to anaemia and low weight at birth in several studies with pregnant women [1-3], although these results are still controversial [4,5]. Even though SMM has been associated to cerebral malaria [6], SMM is only occasionally associated with any clinical manifestation.

SMM is common in endemic countries and its prevalence is comprised between13% and 33% in some areas [7-10]. The higher is the prevalence of macroscopic malaria, the higher is the detected prevalence of SMM [11]. In addition, SMM can be transmitted by infectious mosquito bites, as it has been demonstrated in endemic countries [12].

However, little is known about imported SMM, namely its relevance in terms of frequency, clinical manifestations, or laboratory abnormalities. In countries free of malaria like Spain or Greece, recent autochthonous cases [13-15], suggest the possibility of a malaria re-emergence from undetected imported cases.

This study has two objectives: a) assessing the frequency of imported SMM, and b) describing the epidemiological, laboratory and clinical features of imported SMM.

## Methods

### Study area and design

In Spain, malaria is a reportable disease. Last autochthonous case was notified in 1961 [16] and since then, all reported cases are imported from endemic countries. Hospital Carlos III is a referral unit for tropical diseases at Madrid, Spain. Most patients come by themselves to the emergency unit or are referred from primary care or general hospitals in Madrid. A very small proportion comes from other regions.

A retrospective study based on a review of medical records was performed. The study included those patients older than 15 years and diagnosed with malaria in Hospital Carlos III between January 1st, 2002 and December 31st, 2007. The study was approved by the corresponding Ethics Committee.

Exclusion criteria were: a) Unspecified diagnosis methods; b) Medical records with lack of data (>25% items): epidemiological data (> 5 items), clinical data (> 5 items), or analytical data (> 7 items).

### Malaria, submicroscopic malaria (SMM), and other definitions

A patient was diagnosed with malaria when *Plasmodium* spp. infection could be detected by conventional microscopy and/or using PCR, regardless of the presence of symptoms. A patient was defined as suffering from SMM if he/she produced a positive PCR test even if the examination of thick and thin blood smears by light microscopy was negative. Cases detected by conventional microscopy were considered as microscopic malaria. WHO criteria were followed to identify the severe malaria cases [17].

Cases of malaria were allocated into four groups following the classification criteria published by our study group [18,19]: natives, native-travellers, residents, and travellers. Natives are persons born and living in zones endemic for malaria, that come to a non-endemic zone. Native-travellers are born in endemic zones for malaria, live in non endemic zones (for more than 2 years) and have travelled to endemic zones (country of birth or another). Residents in an endemic zone are people born in non-endemic zones, which have been living in zones endemic for malaria for at least two years. These three groups are considered as semi-immunes. Travellers are defined as people born and living in non-endemic zones that have travelled to zones endemic for malaria (no longer than two years). This last group is considered as non-immune. This classification is more accurate for the purposes of our study than the classical sorting into natives, immigrants, expatriates, and visiting friends and relatives.

### Data collection and laboratory examination

Every patient underwent a complete clinical history and an exhaustive physical examination. They were routinely screened for human immunodeficiency virus (HIV), hepatitis (A, B, C), syphilis, intestinal helminthiasis, or protozoan infestation. The screening for filarariasis (symptomatic or symptom-free) was performed for natives, native-travellers, or residents in endemic zones having travelled to zones endemic for filariae.

For every patient coming from zones endemic for malaria, routine detection techniques for *Plasmodium* included Field’s staining and microscopic examination through thick and thin blood smears. For all patients we always collected one blood sample, independently of the presence of fever. If the sample was negative, a second blood sample was collected only in febrile patients. A semi-nested multiplex malaria PCR [20] served to diagnose or to confirm cases with low parasitaemia. DNA was extracted following the Chelex method with minor modifications. Detection and identification of malarial species were simultaneously performed with a sequence of two SnM-PCRs. The first reaction was expected to yield two products: a band of 231 base pairs (bp) from UNR-HUF produced by the amplification of the small subunit of the human ribosomal gene (positive control), whereas the second reaction yielded a band of 783 to 821 bp from UNR-PLF that should detect the presence of any malaria species of *Plasmodium* spp. In this second reaction, infections with different human *Plasmodium* species yielded products of different sizes. A band of 269 bp indicates an infection by *Plasmodium malariae;* a band of 395 bp evidences a *P. falciparum* infection; a band of 436 bp suggests a *Plasmodium ovale* infection; and a band of 499 bp indicates a *Plasmodium vivax* infection. The mixed infections would show the corresponding bands.

Patients with confirmed malaria (microscopic or submicroscopic) were treated according to the WHO guidelines at the time of the diagnosis. For each case, demographic, clinical, and laboratory data were documented (see Table 1). Anaemia was defined as hemoglobin levels < 13 g/dl (12 g/dl in females); leukocytopenia as total count of white blood cells < 4000 /mm3; thrombocytopenia as total platelets < 150000/mm3; hypoglycemia as glycaemia < 80 mg/dl; and renal failure as creatinine serum levels > 1.2 mg/dl.

**Table 1:** Data collected

	**Data collected**
Demographic data	Age; sex; place of birth; place of residence; last endemic zone visited; dates of travel (relevant for native-traveler and traveler); date of arrival to non endemic zone for malaria (native and native-traveler); date of arrival to endemic zone for malaria (resident in endemic zone); intake of antimalarial chemoprophylaxis (yes/no and drug); adherence to antimalarial chemoprophylaxis (correct vs incorrect).
Clinical data	Presence of symptoms (yes/no); date of onset of symptoms; liver enlargement, spleen enlargement, asthenia, headache, ocular pain, arthromyalgias, vomiting, diarrhea, rash, cough, abdominal pain, jaundice, fever, seizures, hemorrhage, shock.
Laboratory data	Hemoglobin (g/dl); white blood cells (cell/mm^3^); lymphocytes (cell/mm^3^) ; monocytes (cell/mm^3^); platelets (cell/mm^3^); glycaemia (mg/dl); urea (mg/dl); creatinine (mg/dl); lactic deshydrogenase enzyme –LDH- (UI/ml); alanin aminotransferase enzyme -ALT- (UI/ml); total cholesterol (mg/dl).

When a case of malaria lacks of >25% information, it is excluded.

### Statistical analysis

Data were analysed using SPSS package for Windows 17.0 (SPSS, Chicago, IL). For univariate analysis of categorical variables, Pearson’s Chi-square test was used (Fisher test when needed). For continuous data, t-Student test was chosen to compare means between groups, except when the variances of the samples were not homogeneous. In this case (absence of homoscedasticity), the non-parametric Mann–Whitney test was used. A p-value p < 0.05 was considered significant.

## Results

### General features

293 cases of malaria were included in the study. 189 cases (65%) were classified as microscopic malaria and 104 cases (35%) as SMM. Table 2 shows the main features of all cases of malaria by groups. Equatorial Guinea was the main country where infection was acquired (67.91%). Symptomatic cases accounted for 67.57% of the total. Most of the related symptoms were fever (178 cases) and asthenia (175 cases). Associated co-morbidity (mainly other infections) was found in 177 cases (60.41%).

**Table 2:** Characteristics of cases of malaria (total and by group)

**Characteristics of cases of malaria (total and by group)**						
		**Total**	**Traveler**	**Native**	**Native-traveler**	**Resident in endemic zone**
		**n=293; (100%)**	**n=67; (22.86%)**	**n=138; (47.09%)**	**n=14; (4.77%)**	**n=74; (25.26%)**
Age. Mean (SD)		39.66 (14.40)	37.55 (10.45)	41.38 (16.52)	35.39 (9.72)	55.29 (16.68)
Males n (%)		155 (52.9)	42 (62.7)	66 (47.8)	36 (48.6)	11 (78.6)
Median time (days) to diagnosis (range)		11 (0-1544)	8 (0-730)	15 (0-1544)	12 (0-317)	8.5 (0-37)
Antimalarial chemoprophylaxis n (%)		36 (12.3)	21 (31.3)	n.a	15 (20.3)	n.a
Symptomatic n (%)		198 (67.6)	62 (92.5)	56 (40.6)	11 (78.6)	69 (93.2)
Abnormal laboratorial tests	Anaemia	96 (32.8)	11 (16.4)	53 (38.4)	28 (37.8)	4 (28.6)
	Leukocytopenia	50 (17.1)	12 (17.9)	21 (15.2)	12 (18.9)	3 (21.4)
	Thrombocytopenia	126 (43)	32 (47.8)	35 (25.4)	50 (67.6)	9 (64.3)
	Renal failure	31 (10.6)	12 (17.9)	11 (8)	7 (9.5)	1 (7.1)
	Jaundice	9 (3.1)	4 (6)	3 (2.2)	2 (2.7)	0 (0)
Comorbidity n (%)		177 (60.4%)	23 (34.3)	109 (79)	36 (48.6)	9 (64.3)
SMM n (%)		104 (35.5)	13 (19.4)	83 (60.1)	6 (8.1)	2 (14.3)
Plasmodium species	P. *falciparum*	255 (87)	52 (77.6)	123 (89.1)	70 (94.6)	10 (71.4)
	P. *vivax*	13 (4.4)	7 (10.4)	3 (2.2)	1 (1.4)	2 (14.3)
	P. *ovale*	13 (4.4)	4 (6)	7 (5.1)	1 (1.4)	1 (7.1)
	P. *malariae*	6 (2)	3 (4.5)	2 (1.4)	0(0)	1 (7.1)
	Mixed infections	6 (2)	1 (1.5)	3 (2.2)	2 (2.8)	0 (0)
Median of parasitation index -in microscopic malaria- (range)		0.1 (0.1-50)	0.1 (0.1-50)	0.1 (0.1-15)	0.6 (0.1-30)	0.1 (0.1-2.7)

Abbreviations (in alphabetical order). *n.a* Not applicable. *SD* (standard deviation). *SMM* Submicroscopic malaria.

### Analysis by groups

Table 3 shows the epidemiological features of microscopic malaria and SMM. Mean age and sex distribution were similar. Semi-immune group (natives, residents in endemic zones and native-travellers) was more frequent in SMM than in microscopic malaria (p < 0.001). Anti-malarial chemoprophylaxis had been taken in 15.9% of the microscopic malaria cases (30 out of 189), whereas only in 5.8% of the SMM cases (6 out of 104) (p = 0.012).

**Table 3:** Epidemiological features of imported microscopic malaria and SMM

**Epidemiological features by group**					
	**Microscopic malaria**	**Submicroscopic malaria**	**p value**		
**Age. Mean (SD)**	39 (13.9)	40 (15.2)	0.527		
**Male sex. N (%)**	104 (55%)	51 (49%)	0.326		
**Group. N (%)**					
	Traveller (not immune)	54 (28.6%)	13 (12.5%)	< 0.001	
	*Semiimmune*	135 (71.4%)	91 (87.5%)		
		Native	55 (29.1%)	83 (79.8%)	
	Resident in endemic zone	12 (6.3%)	2 (1.9%)		
	Native-traveller	68 (36%)	6 (5.8%)		
**Prophylaxis. N (%)**	30 (15.9%)	6 (5.8%)	0.012		
	Drug				
		Atovaquone-proguanil	3 (1.6%)	4 (3.8%)	
		Doxycycline	3 (1.6%)	0	
		Mefloquine	15 (7.9%)	0	
		Others	9(4.8%)	2 (2%)	
**Comorbidity. N (%)**	102 (54%)	75 (72.1%)	0.002		
	Parasitological infection	25 (24.5%)	55 (73.3%)	< 0.001	
	Filariae	11 (10.8%)	36 (48%)	< 0.001	
	Intestinal helmints	11 (10.8%)	26 (34.7%)	< 0.001	
	Others	16 (15.2%)	30 (40%)	< 0.001	
	HIV	16 (15.8%)	11 (14.7%)	0.831	
	HBV	15 (7.9%)	7 (6.7%)	0.708	
	HCV	14 (7.4%)	9 (8.6%)	0.294	
**Number of different countries of acquisition**	31 (Equatorial Guinea 69.3% (114); Nigeria 5.3% (10); Cameroon 3.7% (7); Mali 3.2% (6))*	15 (Equatorial Guinea 81.7% (85); Cameroon 2.9% (3))*	n.a.		

*SD*: Standard deviation. * Countries representing <2.5% are omitted.

These data may be surprising, but further analysis revealed that only 10% of the patients suffering from microscopic malaria achieved good adherence to chemoprophylaxis (3 cases out of 30), compared to the 66.7% of patients affected by SMM (4 cases out of 6) (p = 0.008). Other parasitic infections were more frequent in the SMM group, but no differences where found concerning infections by HIV, hepatitis B virus (HBV) or hepatitis C virus (HCV). Malaria was caused by *P. falciparum* infection in 171 cases of microscopic malaria (90.5%), and in 90 cases of SMM (86.5%) (p = 0.301; Chi-square test). Infection was caused by other *Plasmodium* species, such as *P. ovale* (8 microscopic malaria cases; 5 SMM cases), *P. vivax* (8 microscopic malaria cases; 5 SMM cases), and *P. malariae* (2 microscopic malaria cases; 4 SMM cases).

We found some relevant differences in clinical characteristics between microscopic malaria and SMM groups (Table 4). Most microscopic malaria cases were symptomatic, while only one out of three cases reported clinical signs in the SMM group. Fever, asthenia and headache were more common in patients with microscopic malaria than in the SMM group. Although tropical splenomegaly was rarely found, it was more frequently noticed in the SMM group (13.3%) than in the MM group (1.8%) (p < 0.011). No severe malaria was found in the SMM group.

**Table 4:** Clinical features of imported microscopic malaria and SMM

**Clinical features by group**			
	**Microscopic malaria**	**Submicroscopic malaria**	**p value**
**Time to diagnosis (days)**	11 (0-730)	12 (0-1544)	0.276 (M)
**Symptomatic**	168 (88.9%)	30 (28.8%)	< 0.001
**Liver enlargement**	17 (9%)	11 (10.6%)	0.0659
**Spleen enlargement**	29 (15.3%)	14 (13.5%)	0.663
**Asthenia**	153 (91.1%)	22 (73.3%)	0.011 (F)
**Headache**	119 (70.8%)	12 (40%)	0.001
**Ocular pain**	39 (23.2%)	3 (10%)	0.103
**Arthromyalgia**	116 (69%)	16 (53.3%)	0.093
**Vomiting**	36 (21.4%)	2 (6.7%)	0.059
**Diarrhoea**	37 (22%)	2 (6.7%)	0.051
**Rash**	1 (0.6%)	1 (3.3%)	0.281 (F)
**Cough**	4 (2.4%)	2 (6.7%)	0.226 (F)
**Abdominal pain**	5 (3%)	2 (6.7%)	0.287
**Fever**	160 (95.2%)	18 (60%)	< 0.001 (F)
**Tropical splenomegaly**	3 (1.8%)	4 (13.3%)	0.011 (F)
**Severe malaria**	13 (6.9%)	0 (0%)	0.005 (F)

Abbreviations (in alphabetical order). *F*: Test de Fisher. *M*: Test de Mann–Whitney.

**Figure 1:**
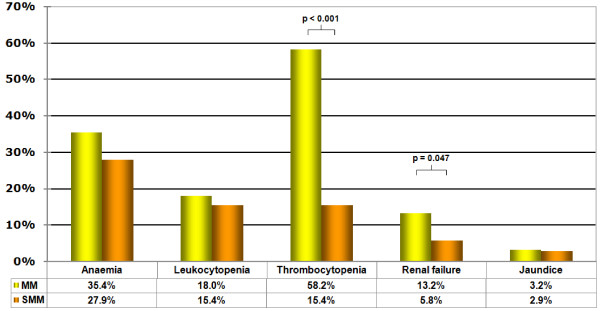
**Comparison of laboratory abnormalities.** MM: Microscopic malaria. SMM: Submicroscopic malaria.

Table 5 shows the analytical values of some blood tests. Comparison of laboratory abnormalities can be seen in Figure 1. More cases of thrombocytopenia (p < 0.001) and renal failure (p = 0.047) were detected in the patients suffering from microscopic malaria than in SMM patients. Anaemia, leukocytopaenia, and jaundice cases were similar in both groups.

**Table 5:** Analytical values of imported microscopic malaria and SMM

**Laboratorial features by groups**			
	**Microscopic malaria Mean (SD)**	**Submicroscopic malariaMean (SD)**	**p value**
**Haemoglobin (g/dl).**	13.1 (1.94)	13.2 (2.04)	0.85
**White blood cell (cell/mm**^**3**^**).**	5,625 (2,008)	6,091 (2,218)	0.06
**Lymphocytes (cell/mm**^**3**^**)**	1,391 (740)	2,176 (824)	< 0.001
**Monocytes (cell/mm**^**3**^**)**	460 (271)	416 (197)	0.131
**Platelets (cell/mm**^**3**^**)**	142,045 (84,860)	216,752 (76,139)	< 0.001
**Glycaemia (mg/dl)**	105 (35)	100 (22)	0.222
**Urea (mg/dl)**	30 (15)	31 (13)	0.497
**Creatinine (mg/dl)**	0.97 (0.38)	0.94 (0.34)	0.539
**LDH (UI/ml)**	482 (236)	419 (189)	0.024
**ALT (UI/ml)**	59 (143)	43 (136)	0.392
**Cholesterol (mg/dl)**	132 (39)	164 (44)	< 0.001

Abbreviations (in alphabetical order). *ALT*: alanine aminotransferase enzyme. *LDH*: lactic dehydrogenase enzyme. *SD*: Standard deviation.

### Symptoms, laboratory abnormalities and co-morbidity

Table 6 shows symptoms and laboratory abnormalities to ascertain whether they are related to malaria or to other co-morbidities.

**Table 6:** Laboratory abnormalities in SMM and comorbidity

**Abnormalities (n)**	**Co-morbidity**	**Comments**
Anaemia (29)	24 (82.8%)	Intestinal parasites (6 cases), tuberculosis (1), thalassaemia-beta (1), chronic kidney disease (1)
Leukocytopaenia (16)	10 (62.5%)	Tuberculosis (1 case), HIV infection (2), HBV infection (1), *Mansonella perstans* (6), and *Salmonella* and *Schistosoma* infection (1)
Thrombocytopaenia (16)	15 (93.8%)	HIV infection (2 cases), *Salmonella* (1)
Renal failure (6)	6 (100%)	Not demonstrated kidney disease.
Jaundice (3)	3 (100%)	HBV infection (1 case), tuberculosis (1), *Salmonella* and *Schistosoma* infection (1)
Liver enlargement (11)	9 (81.8%)	HCV infection (2 cases), HIV infection (3), tuberculosis (1), alcohol-intake (1), drepanocytic anaemia (1), *Salmonella* and *Schistosoma* infection (1)
Spleen enlargement (14)	14 (100%)	HCV infection (2 cases), alcohol-intake (1), tuberculosis (1), *Salmonella* and *Schistosoma* infection (1), drepanocytic anaemia (1), *Mansonella perstans* (5). Not associated (3)
Asthenia (22)	12 (54.5%)	12 different infectious diseases
Headache (12)	6 (50%)	Intestinal parasites (3 cases) acute B hepatitis (1), tuberculosis (1), *Salmonella* and *Schistosoma* infection (1)
Ocular pain (3)	2 (66.7%)	Intestinal parasites (2 cases)
Arthromyalgia (16)	8 (50%)	Acute B hepatitis (1 case), tuberculosis (1), rickettsia infection (1), pneumonia not filiated (1), HCV infection (1), *Mansonella perstans* (2), intestinal parasites (1)
Vomiting (2)	0 (0%)	
Diarrhoea (2)	1 (50%)	*Mansonella perstans*
Rash (1)	0 (0%)	
Cough (2)	1 (50%)	Not associated
Abdominal pain (2)	1 (50%)	Not associated
Fever (18)	11 (61.1%)	Infectious diseases (10), not associated (1)

### SMM in travellers

A total of 13 SMM cases were described in travellers (non immune). A total of 4 patients were symptom-free and did not show laboratory abnormalities. A good adherence to anti-malarial chemoprophylaxis was described in only one of these SMM cases. In the remaining nine symptomatic SMM patients, one suffered from anaemia and one from thrombocytopaenia. For those nine patients, good adherence to anti-malarial chemoprophylaxis was achieved in three cases. When good adherence was achieved the reported symptoms were asthenia (3 cases), arthromyalgia (3), fever (2), headache (1), and cough (1). The symptoms reported when no adherence to anti-malarial chemoprophylaxis was achieved were asthenia (5 cases), fever (4), headache (3), arthromyalgia (3), rash (1), ocular pain (1), diarrhoea (1) and liver enlargement (1).

## Discussion

This study aimed to describe the frequency of imported SMM and its epidemiological, clinical, and laboratory features. Most of the cases of imported malaria reported in Europe are of microscopic malaria and a few reported cases are of SMM. Two main important reasons explain this fact: a) Malaria is suspected when symptoms are present and b) Diagnosis is usually made by microscopic examination but PCR is not routinely used. Examination for SMM has been progressively implemented in the daily care schemes of our hospital, based on several studies showing the high prevalence of SMM in some countries [7,9-11] or on the frequency of symptom-free cases [21,22]. This study could not estimate the prevalence of SMM in the population cared for, because PCR techniques were not systematically performed for all patients (this is not a prospective study). However, the authors believe that SMM is a frequent disease accounting for up to the third of all cases of imported malaria.

Although SMM is a clinical entity usually symptom-free, it can be associated to asthenia, fever or musculoskeletal pain. In addition, anaemia, leukocytopaenia or thrombocytopaenia were reported in more than 10% of the cases. Symptoms and laboratory abnormalities may be linked to other diseases when co-morbidity is present, but sometimes SMM is the only cause. SMM should be envisaged when conventional techniques for malaria detection gave negative results but some clinical or analytical abnormalities remain unexplained in patients coming from zones endemic for malaria.

Negative microscopic tests can produce a positive PCR test in these cases: a) False positive of the PCR technique, or b) Low-density parasitaemias. In low-density parasitaemia, five possibilities have to be considered: 1) Infection in semi-immune people; 2) Anti-malarial treatment; 3) Anti-malarial chemoprophylaxis; 4) First stages of infection; or 5) False negative microscopic method (microscopists with a limited experience in identifying malaria, a real possibility in areas with no transmission).

False positive (or PCR contamination) is a common problem, with rates reported by laboratories between 0.7% and 10% [11,23]. Infection of semi-immune persons is common in endemic countries, as previously mentioned, and has also been described in immigrants [18,24]. Another explanation can be the development of resistance to anti-malarial treatment, as a result of an incorrect management of the treatment or caused by low levels of medication, as well as the “controlled infection¨ in people taking anti-malarial chemoprophylaxis. The early stages of malaria infection can be identified as SMM (even in non-immune people), and may evolve to a “microscopic” infection if no treatment is given.

Data from the WHO Regional Office for Europe have shown that the number of imported malaria cases have not changed significantly in the last ten years (except in France) [25]. Autochthonous malaria cases have been reported in Azerbaijan, France, Georgia, Greece, Italy, Russian Federation, Spain and Ukraine in the same period of time [13,15,25,26]. The possibility of transmission in SMM has been described in endemic zone for malaria [8,12], and presence of a suitable vector in Europe (*Anopheles atroparvus*, *Anopheles claviger* or *Anopheles maculipennis*) has also been reported [27,28]. Since the implementation of molecular tests for all symptom-free individuals coming from endemic area is too expensive, the possibility of a re-emergence of malaria (*Plasmodium vivax*) in Europe can only be speculated.

## Conclusions

SMM is a frequent condition that should be considered when some clinical or analytical abnormalities remain unexplained in a patient coming from zones endemic for malaria. Undetected and untreated SMM and the spreading of competent vectors might be the causes of a re-emergence of malaria in Europe.

## Competing interests

The authors declare that they have no competing interests.

## Authors’ contributions

GRO, PR, MDH, ML and SP attended the patients, collected the data and drafted the manuscript. MS carried out the microscopic examination. JMR carried out the molecular genetic studies. All authors read and approved the final manuscript.

## References

[B1] Mockenhaupt FP Rong B Till H Eggelte TA Beck S Gyasi-Sarpong C Thompson WN Bienzle U Submicroscopic *Plasmodium falciparum* infections in pregnancy in Ghana Trop Med Int Health 2000 5 167 173 10.1046/j.1365-3156.2000.00532.x 10747278

[B2] Arango E Maestre A Carmona-Fonseca J Effect of submicroscopic or polyclonal *Plasmodium falciparum* infection on mother and gestation product: systematic review](in Portuguese Rev Bras Epidemio 2010 13 373 386 10.1590/S1415-790X2010000300002 20857025

[B3] Adegnika AA Verweij JJ Agnandji ST Chai SK Breitling LP Ramharter M Frolich M Issifou S Kremsner PG Yazdanbakhsh M Microscopic and sub-microscopic *Plasmodium falciparum* infection, but not inflammation caused by infection, is associated with low birth weight AmJTrop Med Hyg 2006 75 798 803 17123968

[B4] Saute F Menendez C Mayor A Aponte J Gomez-Olive X Dgedge M Alonso P Malaria in pregnancy in rural Mozambique: the role of parity, submicroscopic and multiple *Plasmodium falciparum* infections Trop Med Int Health 2002 7 19 28 10.1046/j.1365-3156.2002.00831.x 11851951

[B5] Walker-Abbey A Djokam RRT Eno A Leke RFG Titanji VPK Fogako J Sama G Thuita LH Beardslee E Snounou G Zhou A Taylor DW Malaria in pregnant Cameroonian women: the effect of age and gravidity on submicroscopic and mixed-species infections and multiple parasite genotypes AmJTrop Med Hyg 2005 72 229 235 15772312

[B6] Giha HA A-Elbasit IE A-Elgadir TME Adam I Berzins K Elghazali G Elbashir MI Cerebral malaria is frequently associated with latent parasitemia among the semi-immune population of eastern Sudan Microbes Infect 2005 7 1196 1203 10.1016/j.micinf.2005.04.004 15994107

[B7] Roper C Elhassan IM Hviid L Giha H Richardson W Babiker H Satti GM Theander TG Arnot DE Detection of very low level *Plasmodium falciparum* infections using the nested polymerase chain reaction and a reassessment of the epidemiology of unstable malaria in Sudan AmJTrop Med Hyg 1996 54 325 331 10.4269/ajtmh.1996.54.325 8615441

[B8] Shekalaghe SA Bousema JT Kunei KK Lushino P Masokoto A Wolters LR Mwakalinga S Mosha FW Sauerwein RW Drakeley CJ Submicroscopic *Plasmodium falciparum* gametocyte carriage is common in an area of low and seasonal transmission in Tanzania Trop Med Int Health 2007 12 547 553 10.1111/j.1365-3156.2007.01821.x 17445146

[B9] Shekalaghe S Alifrangis M Mwanziva C Enevold A Mwakalinga S Mkali H Kavishe R Manjurano A Sauerwein R Drakeley C Bousema T Low density parasitaemia, red blood cell polymorphisms and *Plasmodium falciparum* specific immune responses in a low endemic area in northern Tanzania BMC Infect Dis 2009 9 69 10.1186/1471-2334-9-69 19460160 PMC2689236

[B10] Touré FS Mezui-Me-Ndong J Ouwe-Missi-Oukem-Boyer O Ollomo B Mazier D Bisser S Submicroscopic *Plasmodium falciparum* infections before and after sulfadoxine-pyrimethamine and artesunate association treatment in Dienga, Southeastern Gabon Clin Med Res 2006 4 175 179 10.3121/cmr.4.3.175 16988096 PMC1570482

[B11] Okell LC Ghani AC Lyons E Drakeley CJ Submicroscopic infection in *Plasmodium falciparum*-endemic populations: a systematic review and meta-analysis J Infect Dis 2009 200 1509 1517 10.1086/644781 19848588

[B12] Schneider P Bousema JT Gouagna LC Otieno S van de Vegte-Bolmer M Omar SA Sauerwein RW Submicroscopic *Plasmodium falciparum* gametocyte densities frequently result in mosquito infection AmJTrop Med Hyg 2007 76 470 474 17360869

[B13] Santa-Olalla Peralta P Vazquez-Torres MC Latorre-Fandos E Mairal-Claver P Cortina-Solano P Puy-Azón A Adiego Sancho B Leitmeyer K Lucientes-Curdi J Sierra-Moros MJ First autochthonous malaria case due to Plasmodium vivax since eradication, Spain, October 2010 Euro Surveill 2010 15 19684 20961517 10.2807/ese.15.41.19684-en

[B14] Florescu SA Popescu CP Calistru P Ceausu E Nica M Toderan A Zaharia M Parola P Plasmodium vivax malaria in a Romanian traveller returning from Greece, August 2011 Euro Surveill 2011 16 19954 21903043

[B15] Danis K Baka A Lenglet A Van Bortel W Terzaki I Tseroni M Detsis M Papanikolaou E Balaska A Gewehr S Dougas G Sideroglou T Economopoulou A Vakalis N Tsiodras S Bonovas S Kremastinou J Autochthonous Plasmodium vivax malaria in Greece, 2011 Euro Surveill 2011 16 19993 22027375

[B16] Clavero Del Campo G The eradication of malaria in Spain](in Spanish Rev Sanid Hig Publica (Madr) 1961 35 265 292 13879820

[B17] Severe falciparum malaria World Health Organization, Communicable Diseases Cluster Trans R Soc Trop Med Hyg 2000 94 Suppl 1 S1 S90 11103309

[B18] Ramírez-Olivencia G Herrero MD Subirats M de Juanes JR Peña JM Puente S Imported malaria in adults. clinical, epidemiological and analytical features](in Spanish Rev Clin Esp 2012 212 1 9 10.1016/j.rce.2011.07.017 22036173

[B19] Ramírez-Olivencia G Herrero MD Subirats M de Juanes JR Peña JM Puente S Imported malaria and HIV infection in Madrid. clinical and epidemiological features](in spanish) Rev Clin Esp 2012 212 10 17 10.1016/j.rce.2011.07.016 22071125

[B20] Rubio JM Benito A Berzosa PJ Roche J Puente S Subirats M López-Vélez R García L Alvar J Usefulness of seminested multiplex PCR in surveillance of imported malaria in Spain J Clin Microbiol 1999 37 3260 3264 10488189 10.1128/jcm.37.10.3260-3264.1999 PMC85544

[B21] Matisz CE Naidu P Shokoples SE Grice D Krinke V Brown SZ Kowalewska-Grochowska K Houston S Yanow SK Post-arrival screening for malaria in asymptomatic refugees using real-time PCR AmJTrop Med Hyg 2011 84 161 165 10.4269/ajtmh.2011.10-0494 PMC3005492 21212221

[B22] Marangi M Di Tullio R Mens PF Martinelli D Fazio V Prato R Angarano G Schallig HDF Giangaspero A Scotto G Prevalence of Plasmodium spp. in asymptomatic African immigrants assessed by nucleic acid sequence based amplification](in Italian Infez Med 2010 18 12 19 20424521

[B23] Wilson SM Detection of malaria parasites by PCR Trans R Soc Trop Med Hyg 1994 88 363 7974692 10.1016/0035-9203(94)90121-x

[B24] Dakić Z Pelemiš M Djurković-Djaković O Lavadinović L Nikolić A Stevanović G Poluga J Ofori-Belić I Milošević B Pavlović M Imported malaria in Belgrade, Serbia, between 2001 and 2009 Wien Klin Wochenschr 2011 123 Suppl 1 15 19 10.1007/s00508-011-0040-x 21826415

[B25] CISID http://data.euro.who.int/cisid/?TabID=281619.

[B26] Armengaud A Legros F Quatresous I Barre H Valayer P Fanton Y D’Ortenzio E Schaffner F A case of autochthonous *Plasmodium vivax* malaria, Corsica, August 2006 Euro Surveill 2006 11 E061116.3 17213553 10.2807/esw.11.46.03081-en

[B27] Bueno Marí R Jiménez Peydró R Malaria in Spain: entomological aspects and future outlook Rev Esp Salud Publica 2008 82 467 479 19039501 10.1590/s1135-57272008000500003

[B28] Bueno Marí R Jiménez Peydró R Could malaria and dengue reappear in Spain? Gac Sanit 2010 24 347 353 10.1016/j.gaceta.2010.02.014 20554081

